# Looking Ahead: Intersecting Influences on Older Gay Men Living With HIV

**DOI:** 10.1093/geroni/igaa057.2561

**Published:** 2020-12-16

**Authors:** Brian de Vries, Gloria Gutman, Tamara Sussman, Shari Brotman, Denis Dube

**Affiliations:** 1 San Francisco State University, San Francisco, California, United States; 2 Simon Fraser University, Vancouver, British Columbia, Canada; 3 McGill University, Montreal, Quebec, Canada

## Abstract

Older HIV-positive gay men live at the intersection of multiple inequalities—with cascading effects on their present and future lives. This qualitative study explored how they plan for their future, with a focus on Advance Care Planning—the process of reflecting/communicating preferences and values for future health and end-of-life care. Seven French-speaking gay men aged 55+ in Montreal, Canada participated in a focus group that was audio-recorded, transcribed and thematically analyzed in four steps. Findings suggest the intersection of sexual orientation and HIV-positive status exacerbated self-disclosure issues; the further addition of age led to preoccupation with day-to-day living and rendered these men vulnerable to social isolation. These tensions not only interfered with their capacities to talk about future care, but also created barriers to thinking about future care. These findings describe the multiple layers and compounding consequences of inequality among older gay men living with HIV.

